# Perceived Stress, Social Support, Emotional Intelligence, and Post-Stress Growth among Chinese Left-Behind Children: A Moderated Mediation Model

**DOI:** 10.3390/ijerph19031851

**Published:** 2022-02-07

**Authors:** Lyuci Zhang, Samsilah Roslan, Zeinab Zaremohzzabieh, Yuqin Jiang, Sumei Wu, Ye Chen

**Affiliations:** 1 Department of Education and Music, Hezhou University, Hezhou 542899, China; zhanglvci1006@gmail.com (L.Z.); jiangyuqin1006@gmail.com (Y.J.); 2 Department of Foundations of Education, Universiti Putra Malaysia, Serdang 43400, Malaysia; z_zienab@upm.edu.my; 3 Department of Education, Guangxi Normal University, Guilin 541004, China; y519@163.com; 4 Mental Health and Education Counselling Center, Guangxi University of Science and Technology, Liuzhou 545006, China; yechen2021upm@gmail.com

**Keywords:** left-behind children, perceived stress, post-stress growth, social support, emotional intelligence

## Abstract

Background: Several previous studies have revealed a negative impact of perceived stress on post-stress growth. Nevertheless, the potential mediating and moderating mechanisms are unclear, particularly for left-behind children in China. Therefore, this study aims to investigate the negative relationship between perceived stress and post-stress growth, the mediating effect of social support, as well as the moderating effect of emotional intelligence in a sample of Chinese left-behind children. Methods: A sample of 837 Chinese students in elementary and middle school was collected for this study. The Perceived Stress Scale, the Social Support Scale, the Emotional Intelligence Scale, and the Post-Stress Growth Scale were employed to examine them. The data were analyzed using SPSS 25.0 software. Results: The results indicate a significant negative association between perceived stress and post-stress growth. Among perceived stress and social support, the former acted as a mediator, while the latter as a moderator. This study sheds light on the post-stress growth of Chinese left-behind children. The findings validated a model of moderated mediation that shows the relationship between perceived stress, emotional intelligence, social support, and post-stress growth. Conclusion: This study confirmed that social support is one of the most important factors among left-behind children, from perceived stress to post-stress growth. Furthermore, the study reveals that emotional intelligence can adjust the relationship between perceived stress and social support to post-stress growth. Therefore, for both family education and school education, the result provides a new direction.

## 1. Introduction

China has undergone an enormous internal migration ever since the 1980s as a result of the rapid economic urbanization process. As a consequence, a new subgroup known as left-behind children has emerged in China, comprising mainly children who have been abandoned in the rural areas while their parents, also called migrant parents, relocate for employment [1]. Typically, left-behind children are youngsters under the age of 18 whose parent(s) relocate for at least six months for work [2,3]. In 2016, rural China had 9.02 million left-behind children, including 870,000 in the eastern region (9.65%), 4.63 million in the central region (51.33%), and 3.52 million in the western region (39.02%) [4]. Parent work migration may be connected with an increased risk of left-behind children experiencing stress in a variety of ways, complicating their socioemotional adjustment. Additionally, past research indicates that left-behind children have increased perceived stress [5] and are associated with increased emotional and behavioral difficulties [6,7,8].

These negative implications, however, do not truly represent the health consequences of parental departure on left-behind children [9], because, as Akhtar [10] noted, “behind these losses lies a revitalized potential for psychic growth” (p. 5). As Tedeschi and Calhoun [11] suggested, the extremely terrifying and painful states evoked by a collection of circumstances that provide considerable obstacles to an individual’s adaptability and comprehension of their world may provide fertile grounds for positive change. They invented the term “post-traumatic growth (PTG)” to refer to the positive changes in an individual’s personality that occur as a result of the survivor’s effort to cope with trauma as well as its psychological implications. PTG can result in enhanced interpersonal connections, more compassion, openness, admiration for life, spiritual development, personal strength, and a revitalized sense of possibility in the world. Zhang and Wang’s research [12] indicated that left-behind children whose interpersonal environment was unfavorable might nonetheless develop PTG, determined by the presence of protective variables that enabled them to withstand the harmful effects of the unfavorable environment.

According to Tedeschi and Calhoun’s cognitive theory [11], social support is a protective factor in the development of post-traumatic growth. A recent study conducted in China discovered that a lack of family and societal support aggravated the psychosocial issues of left-behind children [13,14]. Perceived social support has a strong predictive effect on PTG, according to prior research [15,16]. Joseph and Linley [17] established that social support is a well-known component in lowering stress because it acts as a buffer and may enhance post-traumatic growth-promoting behavior. Prior research established that a perceived or actual support network from nonfamily members such as friends, relatives, neighbors, teachers, and other community members was crucial for these children’s psychological adjustment during their parents’ absence [18,19]. As per the buffer theory, perceived social support acts as a mediating mechanism for individuals, allowing them to buffer the negative impact of the unwanted stimulus, avoid bad emotions, and safeguard their physical and mental health [20,21]. When children who are left behind endure stressful circumstances, the more social support they receive from family, friends, teachers, and classmates, the more post-stress growth they can attain [22].

Additionally, previous research indicated that in the link between social support and adolescent cognition, emotional intelligence can operate as a moderator. Adolescents with high emotional intelligence value the interactive experience of social support more than those with low emotional intelligence, resulting in greater positive cognition and decreased negative cognition [23,24]. Baruah [25] and Li et al. [26] showed that emotional intelligence can indirectly contribute to PTG by assisting with coping. Furthermore, past research revealed that emotional intelligence is critical for stress resistance. Emotional intelligence is a personal trait that demonstrates an individual’s capacity to control their emotions [23] and has been shown to influence PTG. Individuals with a high level of emotional intelligence, according to Li et al. [27], comprehend and evaluate their mental state with great clarity and understand how and when to manage their emotions. These studies showed that disparities in emotional intelligence influence how people react to stress and negative emotions, as well as how they manage stress and control their emotions [28,29]. Therefore, social support and emotional intelligence aid left-behind children in coping with traumatic situations and are indirectly associated with PTG.

To date, numerous studies have investigated the factors that lead to PTG in children and teens who have undergone various types of trauma. There has been no research on post-stress growth and the factors that lead to it in left-behind children thus far. Despite this, few researchers have looked at the potential mediating and moderating mechanisms, and even fewer have studied children who are left behind as a vulnerable group. Against this backdrop, the current study aims to conduct a moderated mediation model to clarify through what experiences (mediating role of social support) and under what conditions (moderating role of emotional support) perceived stress is negatively associated with post-stress growth in a sample of Chinese left-behind children. 

## 2. Literature Review

### 2.1. Perceived Stress and Post-Stress Growth

Perceived stress refers to an individual’s perceptions or ideas about the amount of stress they are experiencing at any particular time or during a specified period. Stress does not always result in negative health repercussions [30]. Chen and Wu [31] discovered that distress and PTG were associated in children and adolescents. PTG is a general term that refers to a positive psychological transformation induced by severely stressful circumstances [11]. More precisely, it refers to a transforming positive experience that occurs before and following the trauma and is a direct result of overcoming difficult life circumstances. According to Tedeschi and Calhoun [11], the PTG experience is significantly reliant on how stressful a situation is rated. Even though various researchers have reported positive [32,33], negative [34,35], or null [36,37] associations between stress and PTG, the nature and directionality of this link in a sample of left-behind children remain poorly understood. Accordingly, this study formulated the following hypothesis:

**Hypothesis** **1.** *There is a negative relationship between perceived stress and post-stress growth*.

### 2.2. The Mediating Role of Social Support

Social support, as a critical social element in child development, is associated with left-behind children’s PTG [38,39]. The term “social support” refers to an individual’s belief that family, friends, and others will provide material aid, emotional support, and knowledge when necessary [40,41]. Chen [42] demonstrated that social capital from family, neighbors, schools, and the community was associated with less delinquent behavior among rural left-behind children. Additionally, Fan and Lu [43] stressed that perceived social support had a large direct effect on the mental wellbeing of left-behind children. According to Folkman and Lazarus’s [44] general model of stress, stress is caused by individuals’ interactions with their environment; if an individual’s living environment is maladaptive or incapable of meeting new demands, stress will manifest or rise. While social support has received widespread attention as a component that reduces stress and increases PTG, a more nuanced picture of the interaction between social support, stress, and PTG in left-behind children must be clearly defined [45]. Social support has been established as a significant predictor of PTG in several studies. Additionally, it has been reported that social support is connected with decreased stress and increased PTG [46]. Nevertheless, it is unknown whether social support can mediate the relationship between perceived stress and PTG in left-behind children. Therefore, the following hypothesis was formulated:

**Hypothesis** **2.** *Social support mediates the relationship between perceived stress and post-stress growth*.

### 2.3. The Moderating Role of Emotional Intelligence 

Emotional intelligence (EI) is a well-known concept, and the studies that focus on EI indicate that it is strongly associated with PTG [26] and perceived social support [47]. Emotional intelligence is defined as an individual’s capacity to identify, use, comprehend, and control emotional information. It is regarded as a critical protective resource [42]. Emotional intelligence can also serve as a protective factor in averting psychological and behavioral disorders in children who have been left behind. It can aid in the development of PTG and more intimate interpersonal relationships. According to the Protective–Protective model [46], the presence of a protective component (e.g., emotional intelligence) enhances the influence of another protective component (e.g., social support) [48] and so reduces stress. As per previous research, emotional intelligence can act as a moderator in the association between social support and adolescent cognition, as well as the association between stress and mental health [23,24]. On the other hand, emotional intelligence can help children avoid danger [49,50]. As stated by Gong and Zhang [51], emotional intelligence can assist individuals in constructively coping with unfavorable external circumstances. Individuals with a high level of emotional intelligence frequently experience less stress and are more likely to have increased PTG [51]. As a result, this study formulated the following hypotheses:

**Hypothesis** **3.** *Emotional intelligence moderates the relationship between perceived stress and perceived social support*.

**Hypothesis** **4.** *Social support mediates the relationship between perceived stress, emotional intelligence, and post-stress growth*.

**Figure 1:**
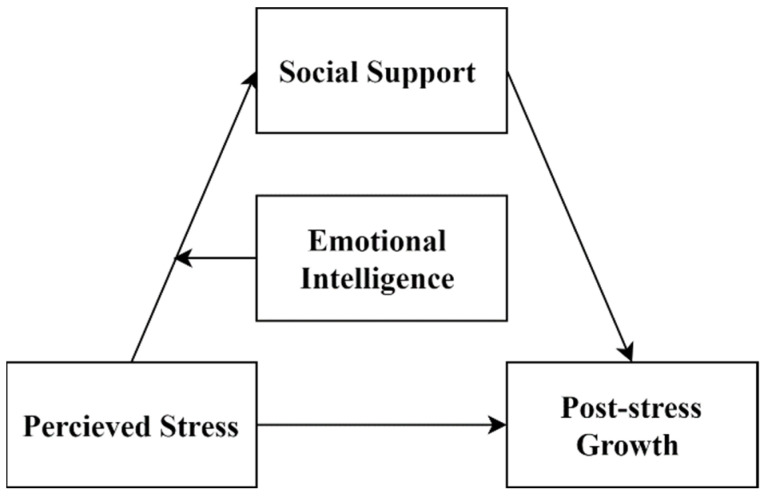
Hypothesized model of the relationships of study variables.

Figure 1 shows a conceptual model depicting the relationships between perceived stress, emotional intelligence, social support, and PTG.

## 3. Materials and Methods

### 3.1. Study Design

A cross-sectional study was used to examine the moderating and mediating effects of social support and emotional intelligence on the association between perceived stress and post-stress growth. This type of design was introduced using a survey to simultaneously investigate and describe the study variables [52].

### 3.2. Participants and Procedures 

A group of 837 rural left-behind children was considered from five elementary and middle schools in Nanning city, Liuzhou city, Qinzhou city, Hezhou city, and Baise city in the Guangxi Zhuang Autonomous Region in China, wherein a large population of migrating laborers reside. The adolescents were from grades eight to twelve. The informed consent of each participant and their guardian was obtained. In addition, accompanied by the class teacher, students answered the questionnaires in the classroom for approximately 30 min, and the questionnaires were collected on-site. Table 1 shows the ratio of boys and girls, parents’ education levels, as well as left-behind status among the various parent migration behaviors.

### 3.3. Measurements

*Perceived stress*. The Chinese version of the Perceived Stress Scale (PSS) has been used in prior research to assess perceived stress in Chinese left-behind children [48]. Chinese scholars Yang and Huang [53] translated and revised the English version of the PSS. They created the Chinese version of PSS based on the realities of a Chinese cultural background, and this scale has a total of 14 items. It is widely used in China and evaluates the perception of the stress individuals are facing over 30 days. The scale contains two factors, namely the sense of tension and the sense of loss of control. The scale adopts a five-point score, and the score of each item is taken as an integer value between 1 and 5. A relatively greater score value shows a greater level of perceived stress. The Cronbach’s alpha coefficient obtained in this study was 0.78.

*Social support*. The youth social support scale was prepared by Yuemei and Dai [54]. This scale is broken down into three different dimensions, namely, subjective support, objective support, and support utilization degree. It contains a total of 17 items. A five-point scoring system is used (inconsistent, somewhat inconsistent, uncertain, somewhat consistent, and consistent), ranging from 1 to 5 points from “inconsistent” to “consistent”. The Cronbach’s alpha coefficient was 0.821.

*Emotional intelligence*. The Chinese version of the Emotional Intelligence Scale (EIS) [55,56] contains 33 items, including emotion perception, self-emotion regulation (regulating emotions), emotion management of others (understanding emotions), and emotion utilization (using emotions to promote thinking). A five-point scale from “completely inconsistent” to “completely consistent” was used. The scale had good reliability (coefficient of 0.84) and validity.

*Post-stress growth*. Qin and Wu’s [57] Post-Stress Growth Scale for Children (PGSC) was adopted. The scale uses a self-assessment method to measure the positive changes of children facing difficulties in the past year. The scale contains 15 items from three different dimensions, namely, interpersonal relationships, problem-solving skills, and life philosophy. A Likert self-evaluation five-point quantitative score was adopted in the scale (0 = “strongly disagree”, 4 = “strongly agree”). The average score of the items was used as an indicator of the individuals’ growth level after pressure. The greater a child’s score on the scale, the higher the individual’s post-stress growth level is. The total internal consistency coefficient of the scale was 0.816.

## 4. Data Analysis 

For the basic sociodemographic parameters, a descriptive analysis, an independent sample *t*-test, and one-way analysis of variance (ANOVA) were obtained. Pearson’s coefficient was then computed for every variable. SPSS 25.0 and Hayes’s [58] PROCESS Macro were used to test the hypotheses. Muller et al. [59] suggested that three statistical steps are needed to establish moderated mediation. Initially, SPSS PROCESS Macro Model 1 was utilized to investigate the overall direct effect of perceived stress on post-stress growth without the moderator or mediator. In the second step, multiple regression was conducted on the mediating effect of social support without the moderator using SPSS PROCESS Macro Model 4 with bootstrapped confidence interval (5000 bootstrap samples). In the third step, Model 7 was specified in the SPSS PROCESS Macro to test the moderated mediation model. While demographic profiles might affect study variables, they were included as controls in the hypothesis testing.

## 5. Results

### 5.1. Common Method Bias Test

In this study, the common method deviation was controlled procedurally by adopting anonymous measurement and partial item reverse measures. Nevertheless, common method bias might exist in the results collected from the self-reported questionnaires [59]. Therefore, based on Kenny’s [59] suggestion, the present study conducted the Harman single factor test to identify the existence of bias. Based on the analysis, 20 factors had eigenvalues greater than 1, and the variance explained by the first factor was 17.608%, which was no more than 40%. Therefore, there was no critical common method bias seen in the study.

### 5.2. Sample Characteristics

The study surveyed 837 left-behind children in China. The sample was composed of 376 (44.9%) males and 461 (55.1%) females, out of which 223 participants (26.6%) were in elementary school and 614 (73.4%) were in middle school. Furthermore, 248 (29.6%) were experiencing father-only migration, 134 (16%) were experiencing mother-only migration, and 455 (54.4%) were experiencing both-parent migration. Independent sample *t*-tests, as well as one-way ANOVA, were employed to assess the demographic variables considered. Table 1 shows the descriptive statistics of the study population along with the results of the analysis. The results indicated that female left-behind children’s post-stress growth was higher than that of male left-behind children (*p* = 0.027). Furthermore, left-behind children’s post-stress growth (*p* = 0.000) from middle schools was significantly higher than those from elementary schools. The post-stress growth of mother-only migration children was significantly lower than that of father-only migration and both-parent migration (*p* = 0.020).

### 5.3. Correlation Analysis

Perceived stress was significantly negatively correlated with post-stress growth (r = −0.105, *p* < 0.01). According to Table 2, social support was positively correlated with post-stress growth (r = 0.573, *p* < 0.01), and emotional intelligence was positively correlated with post-stress growth (r = 0.717, *p* < 0.05).

### 5.4. Testing Social Support as a Mediator

After adjusting the items for gender, grade, and left-behind status, the SPSS PROCESS Macro Model 4 (30) was used to evaluate the mediation model of social support on the relationship between perceived stress and post-stress growth (see Table 3). Perceived stress did not predict post-stress growth significantly (β = 0.02, t = 0.333, *p* > 0.050). Nevertheless, the negative effects of perceived stress on social support were significant (β = −0.514, t = −6.925, *p* < 0.001), as were the positive effects of social support on post-stress growth (β = 0.503, t = 18.908, *p* < 0.001).

The indirect impact of social support on the relationships between perceived stress and post-stress growth was relatively significant (95% CI = −0.338 and −0.183), since 95% CI did not comprise 0. The direct effect was 95% CI = −0.096 and 0.135, since 95% CI included 0. Therefore, the results suggested that the mediation was complete.

### 5.5. Testing for Moderated Mediation

Gender, grade, and left-behind status were selected as control factors depending on the outcomes of the preceding analyses, in agreement with a previous study [60]. The SPSS PROCESS Macro 3.0 Model 7 was employed to estimate the parameters for the two models. In Model 1, the moderating role of emotional intelligence was assessed in the relationship between perceived stress and social support. In Model 2, it was estimated that social support mediated the relationship between perceived stress, emotional intelligence, and post-stress growth. The results of Model 1 (Table 4) displayed that perceived stress was significantly and negatively associated with perceived social support (β = −0.459, t = −7.334, *p* < 0.001). Moreover, perceived stress interacted with emotional intelligence and was negatively associated with social support (β = −0.315, t = −2.556, *p* < 0.05). 

**Figure 2:**
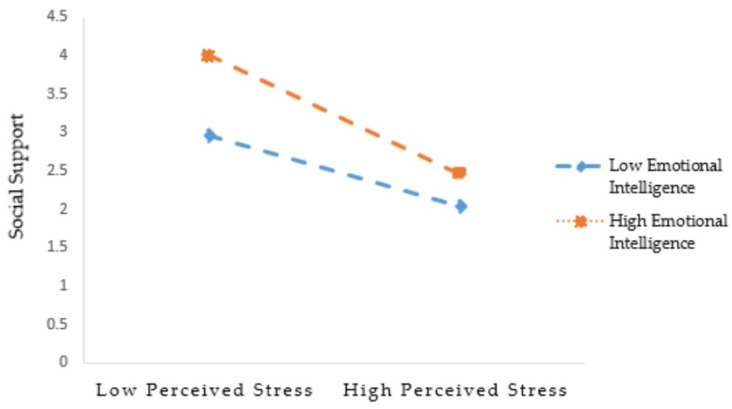
Emotional intelligence moderates the impact of perceived stress on social support.

In addition, to further verify this, a simple slopes test was conducted. More detailed information is shown in Figure 2 and Table 5. The results showed that when emotional intelligence level was high (emotional intelligence = M + 1SD), perceived stress had a strong negative impact on social support (β = −0.622, t = −6.941, *p* < 0.001). When the emotional intelligence level was lower (emotional intelligence = M-1SD), perceived stress still had a negative effect on social support (β = −0.296, t = −3.329, *p* < 0.001); however, the effect was lower than that of the high-emotional-intelligence group. Specifically, left-behind children in both the high- and low-emotional-intelligence groups could significantly improve their social support, as long as they were good at using emotional intelligence after experiencing perceived stress. Nevertheless, this effect was more obvious in those with high emotional intelligence. The results of Model 2 (Table 4) revealed that the indirect effect of perceived stress on post-stress growth via perceived social support depended on the levels of emotional intelligence (β = 0.503, t = 18.908, *p* < 0.001). These results concluded that the relationships between perceived stress and social support were significantly moderated by the levels of emotional intelligence, and the model of moderated mediation for post-stress growth was also significant.

## 6. Discussion

The current study focused on Chinese left-behind children and sought to investigate the moderated mediation roles of social support and emotional intelligence in the association between perceived stress and post-stress growth. The findings indicated that perceived stress was negatively associated with post-stress growth (H_1_ was accepted). Furthermore, perceived social support mediated the relationship between perceived stress and post-stress growth, and that emotional intelligence might act as a moderator between perceived stress and social support, implying that left-behind children with higher emotional intelligence could seek social support more effectively than left-behind children with lower emotional intelligence (these results accepted H_2_ and H_3_). Finally, the results revealed that perceived stress affected post-stress growth via social support with the increment of emotional intelligence (these results accepted H_4_).

First, the present research explored whether left-behind status could affect children’s perceived stress and post-stress growth. This study indicated that the post-stress growth of mother-only migration children was significantly lower than that of father-only migration and both-parent migration. This result was in close agreement with the previous study conducted on 4429 left-behind students [61]. This may be because of Chinese children’s unidirectional obligations to their families and traditional gender role expectations [62]. This view was also supported by the family systems theory and the socialization of emotion model [63]. China’s collectivistic culture promotes interdependence, resulting in emotional experiences that are distinct from those found in Western countries [63]. Chinese children raised in a collectivistic society are more likely to live in harmony with their families, which has an impact on their social emotions and behaviors. 

The results also indicated that female left-behind children’s post-stress growth was higher than that of males. This was consistent with the study of Zhang and colleagues [64]. The explanation for this could be that Chinese left-behind children, in particular female children, tend to acquire positive cultural values in the face of adversity [65]. According to the findings, Chinese female children who adhere to positive cultural views about adversity might develop hope and optimism in the face of adversity, such as parental absence. In addition, the results suggested that the post-stress growth of left-behind children from middle schools was significantly higher than those from elementary schools. The reason could be that middle school students are more mature than elementary school students and they are better at coping with stress and obtaining post-stress growth. Therefore, gender, grade, and left-behind status were chosen as control variables.

Additionally, the results showed that perceived stress and post-stress growth had a significant negative correlation, suggesting that left-behind children with low post-stress growth levels might face higher perceived stress. Lan and others [66] recruited 1210 children from junior high schools and showed that Chinese left-behind children who had undergone more stressful life events were more prone to experience anxiety and nonsuicidal self-injury, both of which are harmful to mental health. In consequence, a high post-stress growth level may help reduce perceived stress. Conversely, it raises perceived stress levels. According to the Stress and Coping Theories [67], when individuals are faced with pressure, they will evaluate the pressure and then make adaptive or maladaptive responses. This study also verified this theoretical model. When left-behind children face stressful events, they will perceive and evaluate the pressure, and then deal with the pressure to produce results. The good result is growth after stress. Based on the Stress and Coping Theories, this study highlighted good results after stress response, i.e., post-growth stress. Therefore, it is very important and necessary to further explore the process of “stress to post-stress growth”.

The results also validated the hypothesis that social support mediated the relation between perceived stress and post-stress growth among left-behind children. This result was consistent with previous research that recruited left-behind children as participants [68]. Therefore, social support—as effective external resources—could enhance post-stress growth after perceived stress, which suggests a novel perspective to validate the relation between perceived stress and post-stress growth among left-behind children. Moreover, sufficient social support could aid in enhancing overall mental health [69] and thus increase psychological adjustment [70]. Maintaining a high level of mental health and psychological adjustment after perceived stress is the performance of post-stress growth. Related studies have found that social support mediated the relationship between perceived stress and mental health [71]. In addition, according to the Stress and Coping Theories [67], individuals can use corresponding coping strategies to cope with stress, and seeking social support from the outside is a very effective way to cope with stress [72] to achieve post-stress growth. Consequently, inadequate social support could hinder post-stress growth; conversely, adequate social support in these situations could encourage people to overcome stress. 

Nevertheless, social support only moderated the relation between perceived stress and post-stress growth. Specifically, the protective effect of emotional intelligence from perceived stress to social support in the group of low emotional intelligence was greater than that in the group of high emotional intelligence. In other words, in the low-emotional-intelligence group, as emotional intelligence increased, individuals were more capable of searching for social support after perceived stress as compared to the high-emotional-intelligence group. Despite this, the moderating impact of emotional intelligence on perceived stress and social support was significantly positive in both high and low groups. This not only showed the moderating role of emotional intelligence, but also highlighted the protective role of social support. Therefore, emotional intelligence and social support are important influencing factors for the post-stress growth of left-behind children. On the one hand, individuals with high emotional intelligence are better at using external social support [73]. On the other hand, according to the Protective–Protective Model [74] there is an interaction between different protective factors in predicting adolescent development. One protective factor can promote the influence of another protective factor on outcome variables. From the model’s point of view, emotional intelligence as a protective factor facilitates the effect of another protective factor (social support) on the outcome variable (post-stress growth). This supports the “Protective–Protective Model”.

## 7. Limitations and Future Research Directions

There are a few limitations in this research that should be addressed. First, this study was cross-sectional, thus making it impossible to draw strong conclusions from the causal association between variables. Therefore, follow-up and longitudinal studies could be performed in the future to address this issue. Second, self-presentation biases might have influenced the associations between the variables because all measurements of the major variables were self-reported. To address these potential biases, future studies would benefit from collecting data from multiple respondents (e.g., mothers and fathers). Third, this study concentrated mainly on the associations between perceived stress, emotional intelligence, social support, and post-stress growth. More studies are needed to explore the influencing factors for the post-stress growth of Chinese left-behind children. 

## 8. Conclusions

This study examined the relationship between perceived stress and post-stress growth in Chinese left-behind elementary and middle school students. Social support was one mechanism by which perceived stress contributed to post-stress growth improvement. In addition, social support entirely buffered the effect of perceived stress on post-stress growth. Moreover, emotional intelligence contributed to the moderating relationship of perceived stress on social support. The findings indicated that social support was a critical component affecting left-behind children’s post-stress growth in the relationship between perceived stress and post-stress growth and that emotional intelligence acted as a moderator in the path between perceived stress and social support.

## Figures and Tables

**Table 1:** Demographics of the study population and association with perceived stress and post-stress growth (*n* = 837).

Variable		N (%)	Perceived Stress (M ± SD)	F/t	Post-Stress Growth (M ± SD)	F/t
Gender	Male	376 (44.9)	3.36 ± 0.34	1.71	3.68 ± 0.68	−2.21 *
	Female	461 (55.1)	3.32 ± 0.31		3.78 ± 0.63	
Grade	Elementary	223 (26.6)	3.31 ± 0.29	−0.492	3.46 ± 0.74	−7.607 ***
	Middle school	614 (73.4)	3.35 ± 0.34		3.83 ± 0.59	
Left-behind status	Father-only migration	248 (29.6)	3.33 ± 0.31	0.52	3.74 ± 0.64	3.93 *
	Mother-only migration	134 (16.0)	3.31 ± 0.31		3.59 ± 0.77	
	Both-parent migration	455 (54.4)	3.34 ± 0.34		3.77 ± 0.62	

The total number of individuals (population) = N; arithmetic mean = M; standard deviation = SD. * *p* < 0.05, *** *p* < 0.001.

**Table 2:** Descriptive statistics and correlation among variables.

No.	Construct	1	3	3	4
1	Perceived stress	1			
2	Social support	−0.221 **	1		
3	Post-stress growth	−0.105 **	0.573 **	1	
4	Emotional intelligence	−0.038	0.535 **	0.729 **	1

** *p* < 0.01.

**Table 3:** Results of the mediation model.

	Model 1 Outcome Variable: SOCIAL Support			Model 2 Outcome Variable: Post-Stress Growth		
	β	SE	t	*β*	SE	t
Gender	0.26	0.048	5.369 ***	−0.029	0.038	−0.759
Grade	0.266	0.049	5.363 ***	0.018	0.039	3.061 **
Left-behind status	0.01	0.027	0.359	0.019	0.021	0.9
Perceived stress	−0.514	0.074	−6.925 ***	0.02	0.059	0.333
Social support				0.503	0.027	18.908 ***
R^2^		0.109			0.338	
F		25.445 ***			84.772 ***	

Regression coefficient = β; standard error = SE; *t*-test values = t; coefficient of determination = R^2^; F-test = F. ** *p* < 0.01, *** *p* < 0.001.

**Table 4:** Moderated mediation model analysis results.

	Model 1 Outcome Variable: Social Support			Model 2 Outcome Variable: Post-Stress Growth		
	β	SE	t	β	SE	t
Gender	0.198	0.041	4.839 ***	−0.029	0.039	−0.759
Grade	0.135	0.042	3.194 **	0.118	0.039	3.061 ***
Left-behind status	−0.021	0.023	−0.921	0.019	0.021	0.9
Perceived stress	−0.459	0.063	−7.334 ***	0.02	0.059	0.333
Emotional intelligence	0.731	0.04	18.313 ***			
Perceived stress × emotional intelligence	−0.315	0.123	−2.556 *	0.503	0.227	18.908 ***
R^2^		0.371			0.338	
F		81.458 ***			84.722 ***	

Regression coefficient = β; standard error = SE; *t*-test values = t; coefficient of determination = R^2^; F-test = F. * *p* < 0.05, ** *p* < 0.01, *** *p* < 0.001.

**Table 5:** The moderating effect of emotional intelligence.

Emotional Intelligence	Effect	SE	t	LLCI	ULCI
M-SD	−0.296	0.089	−3.329 ***	−0.471	−0.122
M	−0.459	0.063	−7.334 ***	−0.582	−0.336
M + SD	−0.622	0.090	−6.941 ***	−0.798	−0.446

Arithmetic mean = M; standard deviation = SD. *** *p* < 0.001.

## Data Availability

The data presented in this study are available on request from the corresponding author.
